# A validated natural language processing algorithm for brain imaging phenotypes from radiology reports in UK electronic health records

**DOI:** 10.1186/s12911-019-0908-7

**Published:** 2019-09-09

**Authors:** Emily Wheater, Grant Mair, Cathie Sudlow, Beatrice Alex, Claire Grover, William Whiteley

**Affiliations:** 1 Centre for Clinical Brain Sciences, University of Edinburgh, Edinburgh, UK; 2 Centre for Medical Informatics, Usher Institute of Population Health Sciences and Informatics, University of Edinburgh, Edinburgh, UK; 3 Health Data Research UK Scotland, Edinburgh, UK; 4 The Alan Turing Institute, British Library, 96 Euston Road, London, UK; 5 Institute for Language, Cognition and Computation, School of Informatics, University of Edinburgh, Edinburgh, UK; 6 Nuffield Department of Population Health, University of Oxford, Oxford, UK

**Keywords:** Radiology, Natural language processing, Brain imaging, Phenotyping, Radiology reports, Stroke

## Abstract

**Background:**

Manual coding of phenotypes in brain radiology reports is time consuming. We developed a natural language processing (NLP) algorithm to enable automatic identification of brain imaging in radiology reports performed in routine clinical practice in the UK National Health Service (NHS).

**Methods:**

We used anonymized text brain imaging reports from a cohort study of stroke/TIA patients and from a regional hospital to develop and test an NLP algorithm. Two experts marked up text in 1692 reports for 24 cerebrovascular and other neurological phenotypes. We developed and tested a rule-based NLP algorithm first within the cohort study, and further evaluated it in the reports from the regional hospital.

**Results:**

The agreement between expert readers was excellent (Cohen’s κ =0.93) in both datasets. In the final test dataset (*n* = 700) in unseen regional hospital reports, the algorithm had very good performance for a report of any ischaemic stroke [sensitivity 89% (95% CI:81–94); positive predictive value (PPV) 85% (76–90); specificity 100% (95% CI:0.99–1.00)]; any haemorrhagic stroke [sensitivity 96% (95% CI: 80–99), PPV 72% (95% CI:55–84); specificity 100% (95% CI:0.99–1.00)]; brain tumours [sensitivity 96% (CI:87–99); PPV 84% (73–91); specificity: 100% (95% CI:0.99–1.00)] and cerebral small vessel disease and cerebral atrophy (sensitivity, PPV and specificity all > 97%). We obtained few reports of subarachnoid haemorrhage, microbleeds or subdural haematomas. In 110,695 reports from NHS Tayside, atrophy (*n* = 28,757, 26%), small vessel disease (15,015, 14%) and old, deep ischaemic strokes (10,636, 10%) were the commonest findings.

**Conclusions:**

An NLP algorithm can be developed in UK NHS radiology records to allow identification of cohorts of patients with important brain imaging phenotypes at a scale that would otherwise not be possible.

## Key messages

- Brain imaging is expensive to perform at scale for research purposes, and automated reading of base images is yet to be developed for most important disease phenotypes. Therefore reading of brain imaging text reports at scale would be useful for research and clinical purposes.
- We developed a natural language processing (NLP) algorithm to identify 24 brain imaging phenotypes in two areas of NHS Scotland which had excellent positive predictive value for cerebrovascular and neurodegenerative phenotypes.
- Use of radiologists’ reports of brain imaging in clinical practice can be useful for cohort development and outcome ascertainment of neurological phenotypes.

## Background

Brain imaging with computerized tomography (CT) and magnetic resonance imaging (MRI) can identify biomarkers of brain pathology that are important for the accurate diagnosis and phenotyping of many neurological diseases. However, brain imaging is expensive, and there are practical constraints to its use for research purposes, particularly in elderly and frail populations. Brain imaging reported by expert radiologists is nevertheless performed very frequently in clinical practice: in NHS England, for example, ~ 700,000 brain MRIs were performed between 2016 and 2017 [1]. Therefore, the reports of brain imaging could aid phenotype definition at lower costs, and be used for large-scale epidemiological studies of volunteers (e.g. UK Biobank) [2], cohorts of patients with disease, cataloguing clinical images, and for system-wide health care quality improvement.

In clinical practice, a radiologist reads a brain image and produces a text report of the findings. However, it is difficult to use these text reports in large scale research studies, because manually coding many thousands of reports is time consuming, and subject to inter- and intra-annotator variation [3]. Many radiology reports are unstructured, despite initiatives to improve this by the Radiological Society of America and other organizations, and therefore are difficult to use with the simplest automated methods of text searching.

One solution is to use natural language processing (NLP) methods to extract information from unstructured text in a radiology report. NLP algorithms can be constructed to identify clinically relevant phenotypes within text and to determine the grammatical relationship between different phrases. Rule-based NLP algorithms can have a high sensitivity (i.e. identify a high proportion of true cases) and high positive predictive value (i.e. a high proportion of those identified are true cases) in clinical records, for example identifying appendicitis, acute lung injury and cancer for use in cohort building, query based case retrieval and quality assessment of neurological practice [4]. For example a US-based study from Partner’s Healthcare identified ~ 6000 cases of cerebral aneurysms and ~ 6000 matched controls with a penalized logistic regression NLP model using radiology reports and other text and routine coding, giving a positive predictive value of 91% for the presence of aneurysms [5]. For the identification of stroke phenotypes, a study of 400 reports from the Mayo Clinic and Tufts Medical Center demonstrated that a rule-based NLP system had an excellent positive predictive value (1.0) for the identification of ‘silent brain infarcts’ and a convolutional neural network had an excellent positive predictive value (0.99) for the identification of white matter disease [6].

We aimed to develop and test an NLP algorithm to extract brain phenotypes from CT and MR brain radiology reports in NHS Scotland. We developed a list of brain phenotypes, primarily related to cerebrovascular disease that could be extracted from reports; determined ground truth in each report by expert review of the text; and developed and validated an NLP algorithm in two different datasets from different regions of NHS Scotland.

## Methods

The datasets used to create the algorithm are available, subject to potential users obtaining the necessary ethical, research and data governance approvals, from Edinburgh Stroke Study (www.dcn.ed.ac.uk/ess) and Health Informatics Centre Services, Dundee (www.dundee.ac.uk/hic/hicservices).

### Datasets

We used two sources of radiology reports to develop and test our automated reading and labelling algorithm: (i) all the brain imaging reports between 2002 and 2014 of participants in the Edinburgh Stroke Study (ESS), a hospital based register of 2160 stroke and transient ischaemic attack (TIA) patients [7] (of whom 1168 could be linked to local radiology reports) and (ii) MR and CT brain reports from NHS Tayside (a different NHS health board within Scotland) performed in unselected in- and out-patients between December 1994 and January 2015 (*n* = 156,619). We received reports stripped of identifiers. We excluded reports of non-brain imaging that were of mixed brain and other body areas, or did not contain a complete radiologist’s report.

We divided each set of reports into datasets for algorithm development (dev) and algorithm validation (test). The ESS data as we received it appeared to be randomized. We reserved the first 500 reports as development data, of which 364 were manually annotated. The remaining 668 reports were further randomized and, of these, 266 were manually annotated to make a test dataset. The Tayside data contained 156,619 reports and we first split this into four equal parts. We used the first part to create manually annotated development data (362 reports) and a randomised version of the fourth part to create manually annotated test data (700 reports). The Tayside data contained a high proportion of ‘normal’ reports, so to enrich it for pathological findings, we used a regular expression search (“blood|bleed|haemor|hemor”) to select reports for the development set. We did not do this for the Tayside test data in order to ensure that it was truly random (of the 700 test reports, only 295 matched the above regular expression).

### Phenotypes of interest, ground truth and agreement between expert readers

**Fig. 1:**
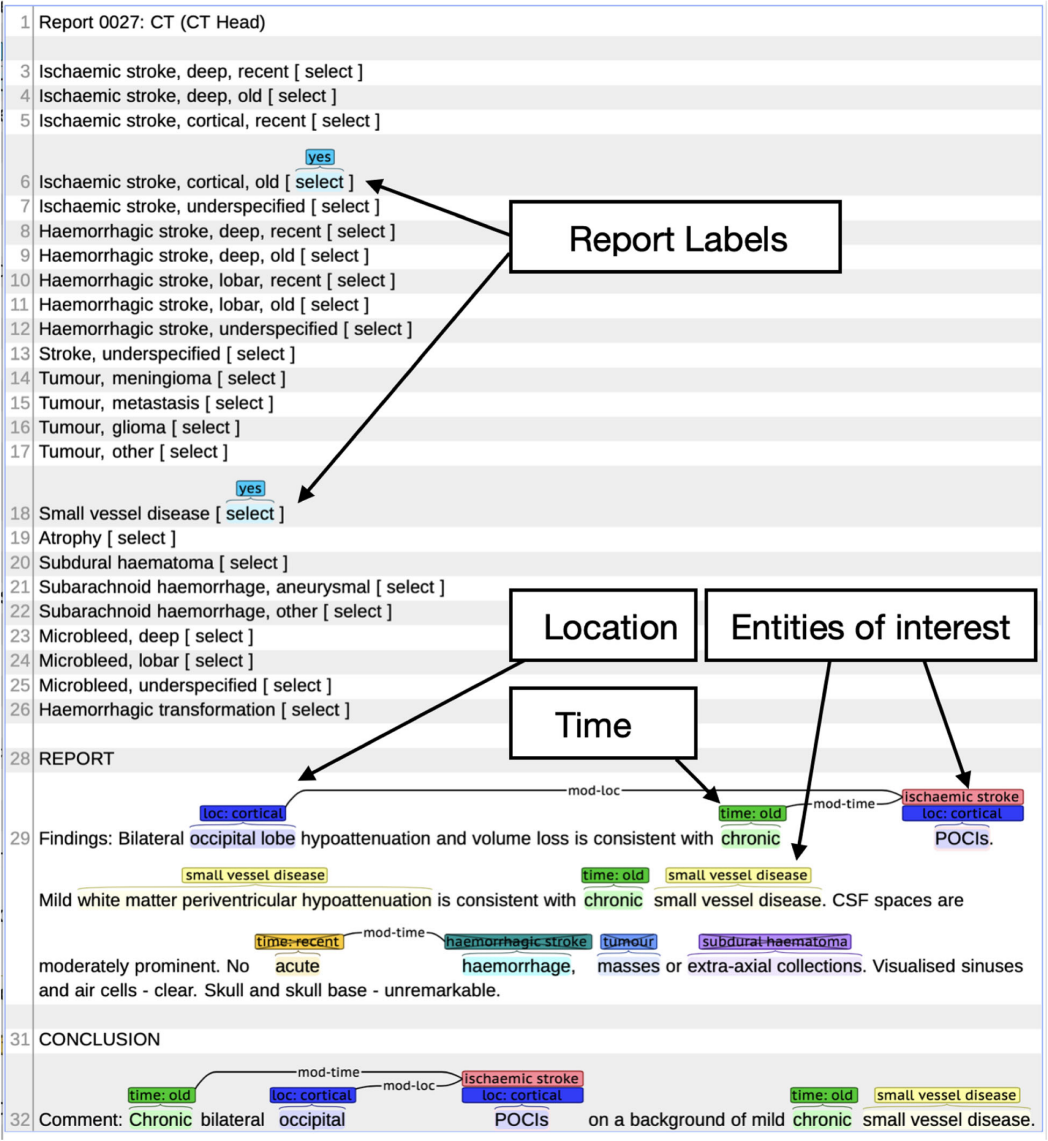
Annotated example report displayed in the Brat annotation tool

Two clinically trained readers (a neuroradiologist and a neurologist, both with specialist expertise in stroke) read 1692 reports and marked up and coded each report with open access annotation software (http://brat.nlplab.org) [8] using the following simple clinically meaningful disease entity and modifier entity annotations in the reports: stroke (haemorrhagic vs ischaemic vs underspecified, deep vs cortical, recent vs old); atrophy (present vs absent); changes of small vessel disease (present vs absent); brain tumours (meningioma, glioma, metastasis, other); subdural hematoma (present vs absent); subarachnoid haemorrhage (aneurysmal or other); microbleeds (deep vs lobar vs unspecified); haemorrhagic transformation of infarct (present vs absent. We combined these annotations into 24 clinically meaningful phenotypes (e.g. old deep ischaemic stroke, recent cortical stroke etc., see Table 1), which the expert readers also identified for each report as annotations on the document level. Stroke types were defined as ‘underspecified’ when it was not possible to assign a location, age or stroke type. Table 1 lists the number of reports, sentences, tokens and annotations for each of the datasets and partitions and also provides detailed counts for each phenotype per partition. An example of an annotated and synthetic but realistic brain imaging report with entity and label (phenotype) annotation displayed via the Brat annotation tool is shown in Fig. 1. A report can be labelled with zero or more phenotypes (min = 0, max = 7, average = 1.4). In the chosen example the report is labelled with two phenotypes (*Ischaemic stroke, cortical, old* and *small vessel disease*).

**Table 1:** Dataset statistics: number of reports, sentences, entity, modifier and phenotype (label) annotation per data set (ESS dev/test vs Tayside dev/test) for annotator 1

	ESS Dev	ESS Test	Tayside Dev*	Tayside Test*
Reports	**364**	**266**	**362**	**700**
Sentences	**3837**	**2855**	**2791**	**3948**
Tokens	**32,229**	**22,842**	**50,522**	**48,519**
Total Entities	**4332**	**2924**	**2997**	**2986**
Disease Entities	2373	1494	1361	1501
Modifier Entities	1959	1430	1636	1485
Total Phenotypes (Labels)	**792**	**518**	**558**	**506**
Atrophy	187	122	90	164
Small vessel disease	245	159	60	145
Stroke, underspecified	24	15	16	< 5
Haemorrhagic stroke, deep, old	2	4	< 5	< 5
Haemorrhagic stroke, deep, recent	2	2	< 5	< 5
Haemorrhagic stroke, lobar, old	4	3	7	< 5
Haemorrhagic stroke, lobar, recent	1	4	< 5	< 5
Haemorrhagic stroke, underspecified	7	10	94	15
Ischaemic stroke, cortical, old	112	61	27	26
Ischaemic stroke, cortical, recent	21	14	19	12
Ischaemic stroke, deep, old	140	85	60	41
Ischaemic stroke, deep, recent	7	4	< 5	< 5
Ischaemic stroke, underspecified	5	12	85	15
Haemorrhagic transformation	1	1	10	< 5
Subdural haematoma	9	6	20	8
Subarachnoid haemorrhage, aneurysmal	1	0	< 5	< 5
Subarachnoid haemorrhage, other	6	6	21	7
Microbleed, deep	2	1	< 5	< 5
Microbleed, lobar	2	1	< 5	< 5
Microbleed, underspecified	0	1	< 5	< 5
Tumour, glioma	0	0	< 5	< 5
Tumour, meningioma	2	4	< 5	< 5
Tumour, metastasis	2	0	22	37
Tumour, other	10	3	21	12

*Small numbers suppressed in the NHS Tayside table due to data governance requirements

We calculated inter-annotator agreement (IAA) between the two clinician annotators for each of the phenotypes in the ESS test dataset (*n* = 266) and a subset of the NHS Tayside test dataset (*n* = 100). We selected a subset of Tayside data for double annotation because we did not have the resources to annotate all test reports twice. The double annotated sample was the final 100 of the 700 randomly selected test reports. We use precision, recall and F1-score for entity annotation agreement because Cohen’s Kappa has been found to be an inappropriate metric for measuring IAA for named entities [9].. We use Cohen’s Kappa for the label (phenotype) annotations [9].

### Natural language processing

**Fig. 2:**
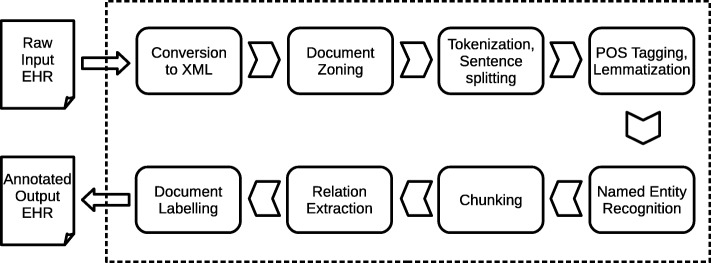
EdIE-R system architecture

We iteratively developed an NLP system to identify the 24 phenotypes in radiology reports. The NLP system, Edinburgh Information Extraction for Radiology reports (EdIE-R), is a staged pipeline process (see Fig. 2), with XML rule-based text mining software at its core [10]. Scan reports are first converted from text format into XML. Each report is then zoned into relevant sections (request, body of report, conclusions) using regular expressions. The text of the body of each report is then split into paragraphs, sentences and word tokens by a tokenization component. This is followed by part-of-speech (POS) tagging where words are labeled with their syntactic categories using the C&C POS tagger [11] in combination with two models, one trained on newspaper text and one on the Genia biomedical corpus [12]. This is followed by a lemmatization step using morpha [13] to analyze inflected nouns and verbs and determine their canonical form (e.g. *bleed* for *bleed*, *bled*, *bleeding* and *bleeds*). All information computed up to this point is the basis for named entity recognition (NER), negation detection and relation extraction. These processes are rule-based and also involve look-up from two manually created domain lexicons (i.e. dictionaries), developed by expert readers’ mark-up of text. These lexicons total around 400 entries though many of these are near duplicates arising from hyphenation and spelling variants (e.g. ‘intracranial’, ‘intra-cranial’, ‘intra cranial’; ‘haemorrhage’, ‘haemorrhage’). The negation detection and relation extraction also rely on an additional chunking step which determines noun and verb phrases in the text. Finally, document-level labels on the patient’s type of stroke or other diseases discussed in the report (phenotypes) are assigned based on the entities and relations present in the text. The rules for this step are a simple mapping from the previous layers of mark-up to the labels. For example, to choose a ‘small vessel disease’ label, the rules need only to check that there is a non-negative small vessel disease entity in either the report or conclusions part of the report. To choose the label ‘Ischaemic stroke, cortical, recent’ there needs to be a non-negative ischaemic stroke entity which is in a location relation with a loc:cortical entity as well as in a time relation with a time:recent entity.

### Assessment of performance

We developed EdIE-R first on the ESS development data set and validated the system on separate, novel reports from the ESS validation data set. We then validated the EdIE-R system in the NHS Tayside dataset, and further developed and improved its performance, before validating it again on unselected unseen NHS Tayside and ESS data. The main further developments were additions to the domain lexicons for named entities that had not previously been encountered, extensions of the rules to recognize negative contexts (e.g. ‘Exclude subdural bleed.’) and fine-tuning of the relation extraction rules.

We report the performance of EdIE-R phenotyping of reports against the reference phenotyping standard of clinical expert reading of reports. For each phenotype, we report sensitivity (proportion of true positive reports identified by EdIE-R), specificity (proportion of true negative reports identified by EdIE-R) and positive predictive value (proportion of EdIE-R positive reports that are true positive). For each measure and phenotype, we calculated 95% confidence intervals using the Wilson method, which generates asymmetrical confidence intervals suitable for values very close to either 100% or 0% [14]. We also report F1-scores which is the harmonic mean of precision (positive predicted value) and recall (sensitivity) and a standard metric used in NLP research.

### Sample size

We based the sample size, *n* = 700, of the validation of the final version of EdIE-R in the Tayside dataset on a sensitivity of 95% for a phenotype of particular interest, old deep ischaemic stroke, with an estimated prevalence of 12% with a 95% Wilson interval width of 10%. The 700 reports were selected at random from the final quarter, *n* = 39,154, of the original Tayside dataset. The development data was selected from the first quarter.

## Results

**Fig. 3:**
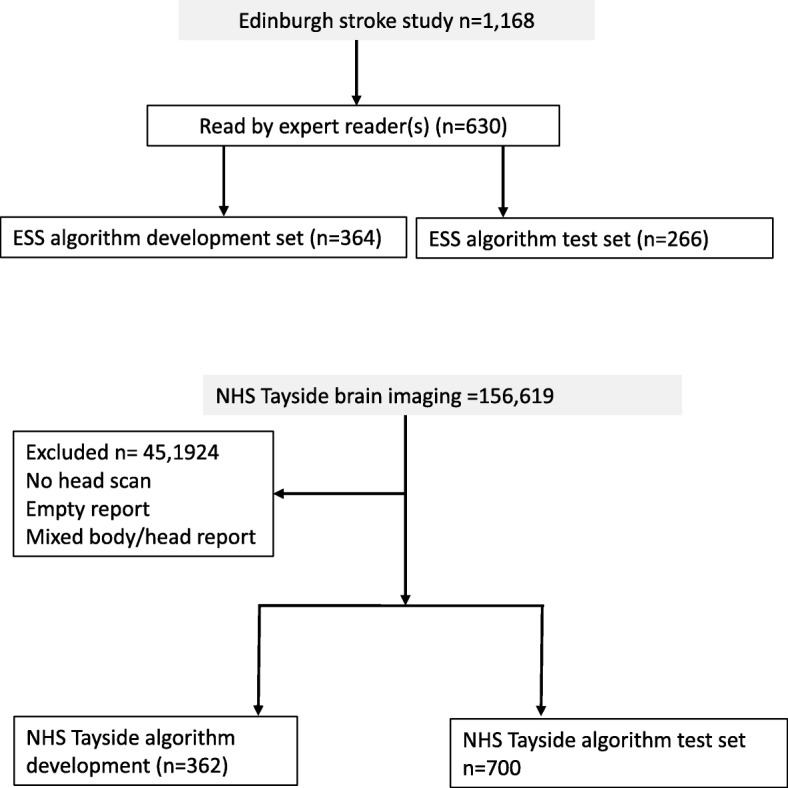
Data flow of patient scan reports from the Edinburgh Stroke Study and NHS Tayside

We first developed the EdIE-R algorithm in 364 reports from the ESS dataset, and internally validated it on 266 further reports from the ESS dataset. We externally validated the algorithm on 362 reports from the NHS Tayside dataset, then further developed the algorithm with different data before a final external validation in 700 reports from NHS Tayside (Fig. 3).

IAA for the entity annotations was high for both subsets. In the ESS subset, we found a precision of 0.96, a recall of 0.98 and an F1-score of 0.97. For the Tayside subset, precision was 0.95, recall was 0.96 and F1 was 0.96 [15]. The agreement between the two expert annotators for all phenotypes was generally excellent in ESS (all Cohen’s κ > 0.95), less so in NHS Tayside (Cohen’s κ 0.39–1.00, see Table 2).

**Table 2:** Inter-annotator agreement measured in Cohen’s Κ between two human annotators in ESS (266 doubly annotated reports) & NHS Tayside (100 doubly annotated reports) for different phenotypes (document-level annotations)

		Κ ESS	Κ NHS Tayside
Atrophy		0.95	0.97
Small vessel disease		0.98	0.97
*Stroke*			
Underspecified	–	0.99	–
Intracerebral haemorrhage	Deep, old	1.00	–
	Deep, recent	1.00	–
	Lobar, old	1.00	1.00
	Lobar, recent	1.00	0.80
	Underspecified	1.00	0.88
Ischaemic stroke	Cortical, old	0.97	1.00
	Cortical, recent	0.98	–
	Deep, old	0.97	1.00
	Deep, recent	1.00	1.00
	Underspecified	0.95	0.71
	Haemorrhagic transformation	1.00	–
*Other intracranial haemorrhage*			
Subdural hematoma	–	1.00	1.00
Subarachnoid haemorrhage	Aneurysmal	–	–
	Other	0.99	0.66
Microbleeds	Deep	1.00	–
	Lobar	1.00	–
	Underspecified	1.00	–
*Tumours*			
Glioma		–	–
Meningioma		1.00	–
Metastasis		–	0.85
Other		0.98	0.39

We developed the NLP algorithm using the ESS dataset, which is enriched for cerebrovascular phenotypes. In unseen ESS validation data, the algorithm had an excellent specificity (≥99%) for all phenotypes and excellent sensitivity for stroke phenotypes, atrophy and small vessel disease (≥95%). However, we identified few cases of haemorrhagic stroke, subdural or subarachnoid haemorrhage, or brain tumours.

We further developed our model in 362 expert-annotated reports in NHS Tayside, and then tested the final EdIE-R model in 700 unselected expert-annotated NHS Tayside reports. The final EdIE-R model had excellent sensitivity, specificity and positive predictive value (all ≥95%) for the following phenotypes: cerebral atrophy, cerebral small vessel disease, and old deep infarcts. The algorithm identified any ischaemic stroke (*n* = 88) with a sensitivity of 89% (95% CI:81–94), positive predictive value of 85% (76–90) and specificity of 100% (0.99–1.00); haemorrhagic stroke (*n* = 23) with a sensitivity of 96% (95% CI: 80–99), positive predictive value of 72% (55–84) and specificity of 100% (0.99–1.00); and any brain tumour with a sensitivity of 96% (95% CI: 87–99), positive predictive value of 84% (73–91) and specificity of 100% (0.99–1.00). For individual stroke and tumour types, the number of patients with any one type was small, and therefore point estimates had wide confidence intervals (see Table 3**).**

**Table 3:** EdIE-R performance on the NHS Tayside test set (n = 700 reports). Small numbers suppressed due to data governance requirements

Label	True Positives (n)	Sensitivity/Recall (95%CI)	PPV/Precision (95%CI)	Specificity (95%CI)	F1 score
Atrophy	159	0.97 (0.93–0.99)	1.00 (0.98–1.00)	1.00 (0.99–1.00)	0.98
Small vessel disease	145	1.00 (0.97–1.00	1.00 (0.97–1.00)	1.00 (0.99–1.00)	1.00
Stroke					
Underspecified	< 5	1.00 (0.34–1.00	0.67 (0.21–0.94)	1.00 (0.99–1.00)	0.80
*Haemorrhagic stroke*					
Any haemorrhagic stroke	23	0.96 (0.80–0.99)	0.72 (0.55–0.84)	1.00 (0.99–1.00)	0.82
Deep, old	–	–	–	–	–
Deep, recent	< 5	1.00 (0.21–1.00)	0.50 (0.10–0.91)	1.00 (0.99–1.00)	0.67
Lobar, old	< 5	1.00 (0.51–1.00)	1.00 (0.51–1.00)	1.00 (0.99–1.00)	1.00
Lobar, recent	< 5	0.75 (0.30–0.95)	1.00 (0.44–1.00)	1.00 (0.99–1.00)	0.86
Underspecified	15	1.00 (0.80–1.00)	0.65 (0.45–0.81)	0.99 (0.98–0.99)	0.79
*Ischaemic stroke*					
Any ischaemic stroke	88	0.89 (0.81–0.94)	0.85 (0.76–0.90)	1.00 (0.99–1.00)	0.87
Cortical, old	24	0.92 (0.76–0.98)	0.92 (0.76–0.98)	1.00 (0.99–1.00)	0.92
Cortical, recent	9	0.75 (0.47–0.91)	1.00 (0.70–1.00)	1.00 (0.99–1.00)	0.86
Deep, old	39	0.95 (0.84–0.99)	0.95 (0.84–0.99)	1.00 (0.99–1.00)	0.95
Deep, recent	< 5	0.50 (0.15–0.85)	0.50 (0.15–0.85)	1.00 (0.99–1.00)	0.50
Underspecified	13	0.87 (0.62–0.96)	0.57 (0.37–0.74)	0.99 (0.97–0.99)	0.68
Haemorrhagic transformation	< 5	1.00 (0.21–1.00)	1.00 (0.21–1.00)	1.00 (0.99–1.00)	1.00
Other intracranial haemorrhage					
Subdural hematoma	6	0.75 (0.41–0.93)	0.86 (0.49–0.97)	1.00 (0.99–1.00)	0.80
Subarachnoid haemorrhage	< 5	0.57 (0.25–0.84)	0.57 (0.25–0.84)	1.00 (0.99–1.00)	0.53
Microbleed	< 5	1.00 (0.34–1.00)	1.00 (0.34–1.00)	1.00 (0.99–1.00)	1.00
Tumour					
Any tumour	52	0.96(0.87–0.99)	0.84 (0.73–0.91)	1.00 (0.99–1.00)	0.90
Glioma	< 5	1.00 (0.44–1.00)	0.60 (0.23–0.88)	1.00 (0.99–1.00)	0.75
Meningioma	< 5	1.00 (0.34–1.00)	1.00 (0.34–1.00)	1.00 (0.99–1.00)	1.00
Metastasis	37	1.00 (0.91–1.00)	0.90 (0.78–0.96)	0.99 (0.99–1.00)	0.95
Other	10	0.83 (0.55–0.95)	0.71 (0.45–0.88)	0.99 (0.99–1.00)	0.77

We tested the potential of the final EdIE-R algorithm to identify patients with particular brain phenotypes in the routinely acquired brain imaging reports from the Tayside region of Scotland. Of 98,036 patients, there was a preponderance of women (54.7%), particularly in the youngest (0–50, 54.9%) and oldest (> 75 yrs. 61.7%) age groups. A minority of patients had been admitted or died with stroke within 30 days of the date of scan (overall 6, 1.5% < 50 yrs., 9.5% > 75 years) (see Table 4). In the 110,695 scan reports of these patients, the most frequent phenotypes were cerebral atrophy (26%), cerebral small vessel disease (13.6%), and deep old cerebral infarcts (9.6%) (see Table 5).

**Table 4:** Demographics of NHS Tayside patients providing reports

Age group (yrs)	Patients (N)	Women (%)	Men (%)	Stroke death or admission within 30 days of scan (%)
0–50	31,860	54.9	45.1	1.5
51–65	19,583	48.8	51.2	6.1
66–75	18,105	50.7	49.3	8.8
Over 75	27,746	61.7	38.3	9.5
Totals	**98,036**	**54.7**	**45.3**	**6.0**

**Table 5:** Proportion of reports with a brain imaging phenotype in NHS Tayside (110,695 reports). Small numbers suppressed due to data governance requirements

	Reports (N)	Percentage of total number of scans (%)
Atrophy	28,757	26.0
Small vessel disease	15,015	13.6
Stroke		
Underspecified	1609	1.5
*Haemorrhagic stroke*		
Deep, old	168	0.2
Deep, recent	397	0.4
Lobar, old	288	0.3
Lobar, recent	415	0.4
Underspecified	5702	5.2
*Ischaemic stroke*		
Cortical, old	4385	4.0
Cortical, recent	1860	1.7
Deep, old	10,636	9.6
Deep, recent	771	0.7
Underspecified	9172	8.3
Haemorrhagic transformation	279	0.3
Subdural hematoma	2272	2.1
Subarachnoid haemorrhage		
Aneurysmal	55	0.1
Other	1381	1.3
Microbleed		
Deep	15	0.0
Lobar	< 10	0.0
Underspecified	19	< 0.1
Tumour		
Glioma	667	0.6
Meningioma	1458	1.3
Metastasis	2621	2.4
Other	4191	3.8

## Discussion

We have developed an NLP algorithm for brain imaging reports in a stroke cohort study in one NHS hospital and validated it with reports from general clinical practice in a second NHS hospital. We have demonstrated excellent diagnostic performance for more common cerebrovascular phenotypes. Although the identification of phenotypes was not perfect, it would have been practically impossible to manually code > 100,000 radiology reports. The ability to code these reports using an NLP algorithm opens the door to using radiology reports to better identify stroke subtype when combined with ICD-10 coded information for outcome ascertainment in large studies such as UK Biobank, for the creation of new in silico cohort studies, or for health care quality improvement [2].

In most research using electronic health records, phenotypes are identified from administrative coding with multiple or single codes (e.g. ICD-10). These codes and combinations have modest to good positive predictive value (> 80%) for all stroke [16]. The addition of NLP summaries of brain imaging report data to administratively coded information, or to NLP processing of medical text, could improve the positive or negative predictive value of stroke identified in EHR. It would also increase the number of stroke that are unspecified (up to 40%) or where stroke type is specified, to allow subtyping of ischaemic stroke types where this is not available (for example in our center, codes for lacunar stroke are rarely used). In addition, some asymptomatic findings that are not routinely coded consistently (e.g. changes of cerebral small vessel disease) could be identified. This could be particularly useful for deriving neurological phenotype at scale from health records in large scale cohorts such as UK Biobank (*N* > 500,000), [2] and the NIH-funded All of Us study (planned *N* = 1,000,000 https://allofus.nih.gov).

The performance of our algorithm differed to a modest degree in the ESS dataset which was enriched for cerebrovascular phenotypes and NHS Tayside from general radiology practice. This is probably accounted for by the differences in language used across different radiology departments; and the different prevalence of findings in different datasets with higher prevalence leading to greater positive predictive value.

The high IAA scores indicate that the annotation tasks were well-defined. In previous work we have demonstrated the impact annotation has on NLP performance [17]. The same is true for this task, the better and more defined the annotation the easier it is to extract the same information automatically. Before doing the annotation, the experts carried out some pilot annotation on paper and we decided on a set of rules for what to annotate. In some cases, the high IAA can also be attributed to the consistent use of medical terms in this domain. While there is some variation, certain diseases and symptoms are described with widely used and well-known expressions and it is straightforward for experts to identify them in text.

The high accuracy of the rules in our system results largely from the topic-focused nature of the radiology reports and the fact that the language is restricted and conventionalized, with only a limited number of ways in which a phenomenon tends to be described. Several of the system errors arise from unexpected ways of phrasing concepts, for example, the entity subarachnoid_haemorrhage is most frequently expressed as ‘subarachnoid haemorrhage’, ‘subarachnoid blood’ or ‘SAH’, and the system failed to recognize it in a report where it was described as ‘blood in the subarachnoid spaces’. This is the kind of problem that unavoidably occurs when test or run-time data contains unseen examples, i.e. ways of expressing concepts that have not been seen in the training/development data. This is true of both supervised machine-learning and rule-based systems. At the final stage of assigning labels to documents, labels will be missed if the relevant named-entities or relations have been missed (false negatives). Errors at this stage also arise from false positives from NER, relation detection and negation detection. Clear cases of time and location relations (e.g. ‘Right frontal chronic haemorrhage’) are straightforward to detect but the system can make errors in linking a time or location to an entity because it does not take sentence-structure into account. For example, in the sentence ‘I suspect this reflects redistribution of the original haematoma rather than new blood.’, the time entity ‘new’ was wrongly linked to the haemorrhagic stroke entity ‘haematoma’. With regard to negation, clearly stated absence of a phenomenon (e.g. ‘No metastases’) are reliably detected but in cases where the annotator has marked an entity as negative in an unclear context (e.g. ‘diffusely sclerotic metastases are much less likely’), negation detection can fail and this can lead to a false positive in the labelling.

The phenotypes we have chosen are those that are relevant for epidemiological and clinical researchers. There are limits to the detail in reports in clinical practice, hence we chose to identify phenotypes that we thought would be possible to code. In different settings (for example hyperacute stroke services), there may be more detail in individual reports to identify other phenotypes such as vessel occlusion. Further work is needed to enrich our samples for less common phenotypes (for example individual types of haemorrhagic stroke or tumours); to determine the diagnostic contribution of NLP alongside structured, coded data (e.g., ICD-coded hospital admissions or Read-coded primary care consultations); to compare the performance of NLP coding of reports against research-grade reads of images; and to implement these algorithms within NHS systems.

In terms of portability and generalisability of our NLP system, we have shown that EdIE-R is robust in phenotype labelling performance when porting it from one dataset to another (ESS to Tayside). The same holds true for named entity recognition (NER) on the same data. While we spent some effort on fine-tuning the system on the new development data, this did not take a substantial amount of time [15]. We would expect a high level of performance when running the EdIE-R system over new brain imaging reports. To port the system to a new type of medical text, e.g. radiology reports for a different disease or body part, or to pathology reports, we would require a new lexicon and would need to adapt some of the rules. This is not an insignificant amount of effort and requires input from domain experts. Instead we could use machine learning methods but would then require more training data (annotated by domain experts), as well as time to fine-tune parameters or features, in order to reach or exceed the same level of performance as the rule-based method.

## Conclusions

In summary, we have demonstrated that an NLP algorithm can be developed with neuroradiology reports from the UK NHS radiology records, allowing identification of cohorts of patients with important cerebrovascular phenotypes at a scale that would otherwise not be possible.

## Data Availability

Brain imaging reports are available, after appropriate approvals have been obtained from an ethics board (a UK-based ethics committee) or data governance board (NHS Tayside) covering the use of these data. The NLP algorithms are available on application to the authors.

## Abbreviations

CI: Confidence interval
CT: Computerised tomography
EdIE-R: Edinburgh Information Extraction for Radiology
ESS: Edinburgh stroke study
IAA: Inter-annotator agreement
MRI: Magnetic resonance imaging
NER: Named entity recognition
NHS: National Health Service
NLP: Natural language processing
POS: Part-of-speech
PPV: Positive predictive value
TIA: Transient ischaemic attack
